# The potential of pumice as a litter material and its influence on growth performance, carcass parameters, litter quality traits, behavior, and welfare in broiler chickens

**DOI:** 10.1007/s11250-024-03979-z

**Published:** 2024-04-18

**Authors:** Mustafa Duman, Ahmet Şekeroğlu, Brian Tainika

**Affiliations:** 1 Department of Laboratory and Veterinary Health, Bor Vocational School, Niğde Omer Halisdemir University, Niğde, 51240 Turkey; 2 Department of Animal Production and Technologies, Faculty of Agricultural Sciences and Technologies, Niğde Ömer Halisdemir University, Niğde, 51240 Turkey

**Keywords:** Acidic pumice, Basic pumice, Broiler, Litter material, Performance, Welfare traits

## Abstract

This study evaluated the possibilities of pumice (light stones) as litter material in broiler production. Experimental treatments included wood shavings (WS), acidic pumice (AP), and basic pumice (BP) alone, and in combination; wood shaving + acidic pumice (WSAP) and wood shaving + basic pumice (WSBP) in a ratio of 1:1. Two trials were performed, one in summer, and the other in winter. Each trial involved 750 mixed-sex Ross (308) broilers. Also, there were 15 replicate pens with 50 broilers and a stocking density of 12.5 birds/m^2^ for each pen at the beginning of each trial. Performance, litter quality, carcass parameters, body and leg abnormalities, body temperature, fear and stress responses, proportional asymmetry, and some behavior expressions were investigated. The litter treatment influenced the final live body weight, litter moisture, ammonia concentration, footpad dermatitis, hock burn, breast blister, hot carcass yield, heart, liver, spleen, abdominal fat, wing and neck ratio, breast and back cleanliness, and the expression of dust bathing and foraging behaviors (*P* < 0.01; *P* < 0.05). Furthermore, there was a seasonal effect on live body weight, feed conversion ratio, livability, litter pH, 42-day litter moisture, hot carcass yield, back cleanliness, footpad dermatitis, hock burn, footpad temperature, heterophil-to-lymphocyte ratio, and expression of pecking behavior (*P* < 0.01; *P* < 0.05). It is suggested that acidic pumice stone alone or in a mixture with wood shavings could be used as a reliable litter material, alternative to wood shavings.

**Supplementary Information:**

The online version contains supplementary material available at 10.1007/s11250-024-03979-z.

## Introduction

Environmental factors greatly impact the performance, health, welfare, and behavioral expression of broilers (Tainika et al. 2023; Wilcox et al. 2023). In the poultry industry, litter is among the important environmental factors affecting performance, welfare criteria, and product quality (Wilcox et al. 2023). Factors including economic advantage, availability, water holding capacity, potential hazards, and contamination are among the considerations when choosing a litter material (Şekeroğlu et al. 2013; Gerber et al. 2020).

Previous authors have reported the influence of different litter materials on some performance and litter quality variables of broilers (Sarıca and Cam 2000; Garcês et al. 2013; Durmuş et al. 2023; Şen et al. 2023). Other studies determined that litter types varied in the occurrence of welfare issues such as footpad dermatitis (Mendes et al. 2011; Durmuş et al. 2023). Meanwhile, behavioral studies identified that litter materials differed in attractiveness for broilers to express litter-directed behaviors including ground scratching, foraging, and dust bathing (Villagrá et al. 2014; Holt et al. 2023).

Wood shavings are the most preferred litter material in broiler production but other uses of wood shavings have made the availability of this material difficult, resulting in increased supply costs (Toledo et al. 2019; Gerber et al. 2020). Therefore, recent research has mostly focused on determining cheap and easily supplied litter materials as alternatives for wood shavings. Besides wood shavings, examples of litter materials that have been tested in broiler houses include sawdust, rice husk, corncob shavings, hazelnut husk, wheat hay and straw, shredded paper, sand, limestone, and cotton residues (Grimes et al. 2006; Şekeroğlu et al. 2013; Toledo et al. 2019).

However, there aren’t any studies in the literature about the possible use of pumice as a litter material in broiler houses. Pumice (light stones) are formed as a result of volcanic events, they have a hollow, spongy structure, and are abundant in acidic and basic forms worldwide (Tanyıldızı and Gökalp 2023; Wang et al. 2023). Pumice is used in many industrial sectors in the world: construction, textile, agriculture, chemistry, and other industrial and technological fields (Tanyıldızı and Gökalp 2023). Pumice is a cheap, easy-to-haul material, rich in silica oxides, and has quite a high water absorption capacity (Tanyıldızı and Gökalp 2023). Like natural zeolites, pumice with quite high water holding capacity (about 53% of its weight) can reliably be used as a litter material in broiler housings. According to Yücel et al. (2020), acidic pumice is white and off-white, its hardness is 5–6 based on the Mohs scale, and its density is 0.5-1 gr/cm3. Basic pumice is dark, brownish, or blackish, heavier, its hardness is 5–6, and its density is 1–2 g/cm3.

The determination of appropriate and affordable litter resource alternatives for wood shaving remains significant in the poultry industry, especially where broiler production is increasing with the increasing demand for chicken meat. Therefore, in this study, the effects of wood shavings, acidic pumice, and basic pumice alone and in combination on broiler growth performance, litter quality, carcass traits, behavior, and welfare were investigated.

## Materials and methods

### Experimental area, animal materials, and animal husbandry

The research was carried out in the broiler barn at the Poultry Unit of the Department of Animal Science, Faculty of Agriculture, Gaziosmanpaşa University.

The animal material of the experiment consisted of mixed-sex broiler chicks (Ross 308), which were obtained from a private commercial breeder. On the day of arrival corresponding to the start of the trials, the day-old chicks were weighed to ensure that the average chick weight was similar in the replicate pens. The wing-tagged chicks were distributed to 15 pens of 2 × 2 m in size (5 litter treatments × 3 replicates) according to the study plan. 50 birds with an equivalent of 12.5 birds/m^2^ were allotted to each replicate pen. There were 150 chicks in each litter treatment, 750 in each trial (summer; 09 July – 19 August and winter; 26 December – 05 February), and a total of 1500 chicks. The number of males and females in each litter treatment replicate pen at the beginning and at the end of the experiment (42 days of age) both in the winter and summer season trials are indicated in Supplementary Table 1.

**Fig. 1:**
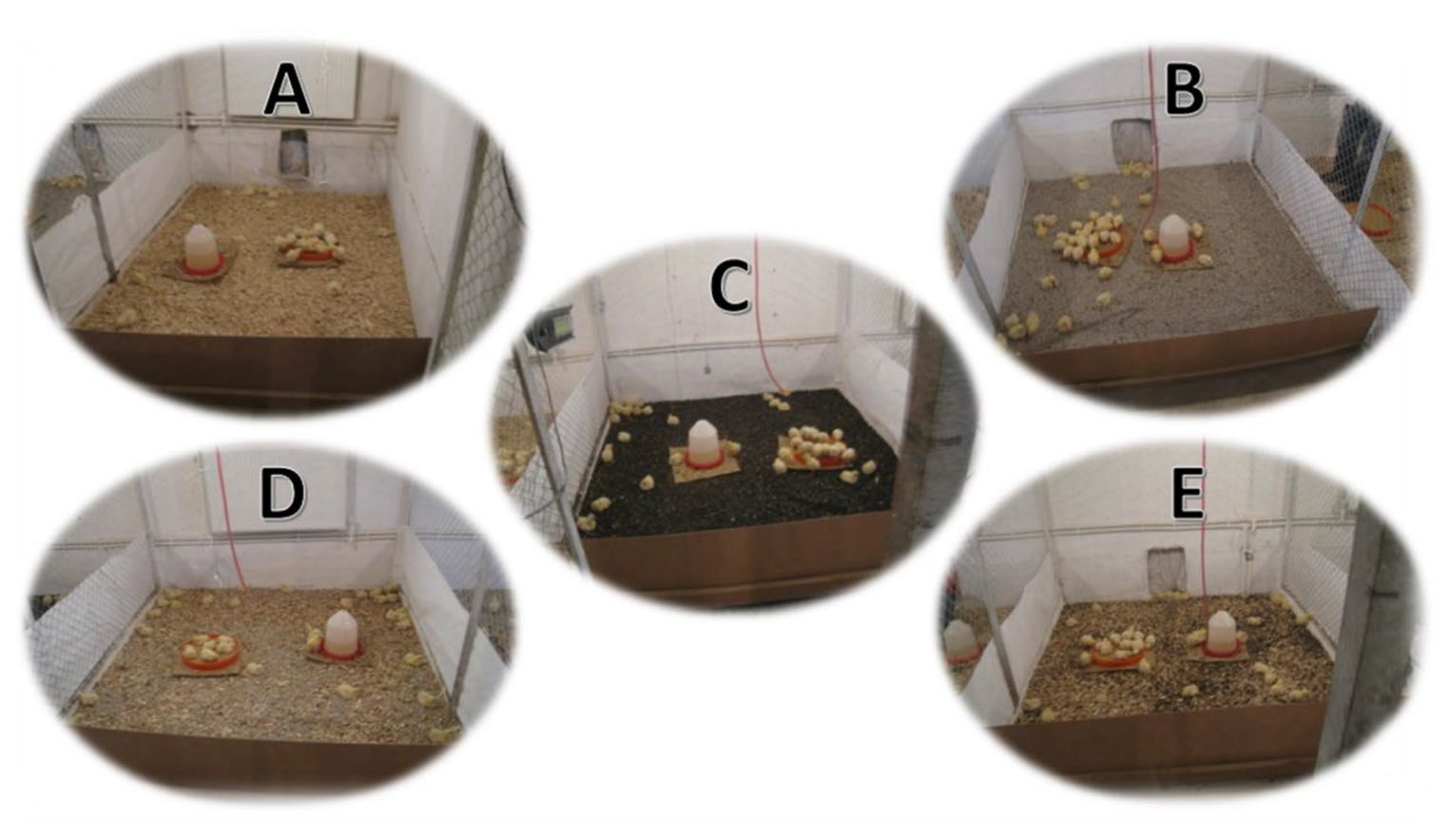
Experimental litter treatments (**A**: Wood shavings, **B**: Acidic pumice stone, **C**: Basic pumice stone, **D**: 50% wood shaving + 50% acidic pumice stone, **E**: 50% wood shaving + 50% basic pumice stone)

The litter treatments in each trial involved wood shavings (WS), acidic pumice (AP), and basic pumice (BP), and the mixtures: wood shavings + acidic pumice (WSAP), and wood shavings + basic pumice (WSBP) in a ratio of 1:1. The litter treatments in each trial are shown in Fig. 1.

The average litter thickness for all litter groups was 5 cm. To prepare mixtures consisting of different litter materials, each replicate pen of 4 m^2^ was divided into two equal parts. Thereafter, each litter material was laid in the corresponding section until it was 5 cm deep and consequently, mixed thoroughly.

Standard concentrate feeds purchased from a commercial enterprise were used as feed material throughout the trial. Chick starter feed: between 0 and 14 days (d), broiler grower feed: between 15 and 35 d, and broiler finisher feed: between 36 and 42 d. The general composition of the feeds used in the seasonal trials is given in Table 1.

**Table 1:** General nutrient composition of feeds used in the summer and winter trials

Nutrient composition	Summer			Winter		
	Chick starter feed	Broiler grower feed	Broiler finisher feed	Chick starter feed	Broiler grower feed	Broiler finisher feed
Crude protein, %	22	20	18	23	21	18
Crude cellulose, %	5	5	5	4	4	4
Crude ash, %	9	9	9	8	8	8
Calcium, %	1-1.5	0.8–1.2	0.9–1.5	1-1.5	0.8–1.2	0.9–1.5
Phosphorus, %	0.50	0.40	0.40	0.5	0.45	0.40
Lysine, %	1.2	1.1	1.0	1.3	1.1	1.0
Methionine, %	0.6	0.5	0.4	0.6	0.5	0.4
Metabolic energy, Kcal/Kg	2900	3050	3200	3050	3150	3200

The study barn was naturally ventilated and had tube feeders and automatic hanging drinkers. Electric heaters ensured the heating of the barn. From the first until the final day (42 d) in both trials, the temperature and humidity values inside the barn were recorded per hour using data loggers hanging at the back level of the birds. The weekly ambient temperature and humidity in the two trials per treatment group are shown in Supplementary Tables 2 and 3, respectively.

From the day of the arrival of chicks to the final day of the trials, a photoperiod (light to dark, L:D) of 24 L:0D was ensured.

### Data collection during each trial (summer and winter trials)

#### Performance traits

##### Weekly live weight (LW)

was determined by weighing the birds individually using a scale with an accuracy of 0.1 g.

##### Weekly live weight gain (LWG)

was determined by subtracting the live weight of the birds at the time of weighing from the live weight of the previous week. The LW and LWG were later determined by pen replicate.

##### Weekly feed intake (FI)

was determined by subtracting the remaining leftover feed at the end of the week from the total feed given during the week on a pen basis. The given and remaining feed weights were determined by weighing with a scale with an accuracy of 0.1 g.

##### Feed conversion ratio (FCR)

was calculated by using LWG and FI as ((g feed / g LWG)) and then determined cumulatively (0–21 days and 0–41 days of age) by pen replicate.

Dead animals were recorded daily, and livability on a pen basis was calculated from these data at 42 days of age. However, it is emphasized that the FI and FCR of dead birds were not corrected.

#### Litter quality traits

##### Litter moisture

At the beginning of the trials and on a weekly basis, samples taken from 5 different parts of each replicate pen (4 corners and the center) and approximately 3 cm below the litter surface were thoroughly mixed and the litter moisture was determined by dry matter analysis (İpek et al. 2002). Approximately 5 g of the mixed sample was weighed into glass dry matter containers with lids and dried at 105 °C until it reached a constant weight (12 h). Moisture content was determined based on the weight loss value of the samples.

##### Ammonia (NH_3_) concentration

was based on the average of the values measured at the nostril level of birds from 5 different locations in each pen per week with an ammonia meter device (PGas-24 NH3/CO2) (Akşit et al. 2000).

##### Litter pH

Litter samples randomly taken from different parts of each pen (4 corners and center) were mixed weekly. 20 g of the sample taken from this mixture was mixed thoroughly with 30 mL of distilled water in a sterile container and left for 1–2 min. Thereafter, the pH of the litter was measured and recorded with a pH meter (Pope and Cherry 2000).

##### Litter temperature (°C)

was determined weekly based on the average of the values obtained from 5 different parts of the litter for each treatment replicate pen (4 corners and center) in the morning (7:00 a.m.), afternoon (2:00 p.m.), and evening (9:00 p.m.) during the day. The litter bottom and surface temperatures were determined with a digital thermometer and an infrared thermometer, respectively (Bilgili et al. 1999).

##### Behavioral expression

was determined by instant observation of birds in each replicate pen in the morning (8:00–12:00) and afternoon (1:00–5:00 p.m.) on 2 consecutive days in the 3rd, 4th, 5th, and 6th weeks of the trials. In each observation session (6 min), the number of birds exhibiting the behaviors shown in Table 2 was recorded at 2-minute intervals. (Bayram and Özkan 2010). Behavior observation was performed by a Ph.D. researcher with vast experience and training in behavioral studies in poultry.

**Table 2:** Behaviors observed in broilers during the trials

Behavior	Description
Dust bathing	While lying on the ground, the broiler is kicking loose litter onto his/her feathers and throwing it over his/her body using the wings and full body movement
Ground scratching (foraging)	Broiler scratching feet backward in the litter followed by ground pecking
Pecking objects	Broiler pecking objects other than feed

#### Welfare traits

##### Footpad dermatitis (FPD)

was examined on the 21 and 42 d on all birds in each pen replicate. A 4-point scoring scale was used (Welfare Quality 2009). Briefly, score 0: no lesions, score 1: lesions less than 5% of the footpad surface, score 2: lesions less than 25% of the base area, score 3: lesions greater than 25% and ulcerated.

##### Hock burn (HB)

Before slaughter (42 d), all birds in each pen replicate were visually examined for hock burn as described by Welfare Quality (2009). According to the degree of inflammation, the degree of hock burn was scored as 0: no lesion, 1: small inflammation, 2: medium-sized inflammation, and 3: large and ulcerated inflammation.

##### Valgus/varus (VV) and twisted leg (TL)

were scored before slaughter (42 d) on all birds in each pen replicate, using a 3-point scoring scale. 0: no opening or narrowing of the hock (less than 5 degrees), 1: mild opening or narrowing of the hock (angle between 5 and 20^o^), 2: intermediate opening or narrowing of the hock (angle between 20 and 40^o^), 3: intense opening or narrowing of the hock (more than 40 degrees). Broilers that could not walk due to these problems were scored as having/not having a twisted leg problem (Sørensen et al. 2000).

##### Breast blisters (Bb)

After slaughter, all the carcasses were visually observed for breast blisters. According to the degree of inflammation, deformation in the breast was graded as 0: no lesion, 1: small and light-colored lesions, and 2: large, dense, and dark-colored lesions (Nielsen 2004).

##### Body region temperatures (°C)

Rectal, footpad, and breast surface temperatures of 6 randomly selected birds (3 males, 3 females) from each replicate pen were recorded weekly starting from the 14 d. Rectal temperature was measured by inserting a digital thermometer approximately 3 cm into the cloaca. The digital thermometer was kept in the cloaca of the birds until the temperature increase stabilized. Footpad and breast temperatures were expressed as the average of the values measured using an infrared thermometer from 3 different parts of the respective regions.

##### Feather cleanliness score (FCS)

Scoring of feather cleanliness was performed on the day 21 and 42 on the back and breast regions of all broilers per pen replicate. A 3-point scoring scale was used; 1: slightly dirty, 2: medium dirty, and 3: very dirty according to the cleaning status (Welfare Quality 2009).

##### Duration of tonic immobility (TI, seconds)

A total of 90 broilers; 18 (9 males and 9 females), from each litter type were tested. Briefly, each bird was laid on its back in a rectangular short-open container, with the head hanging in space, held by gentle pressure on the breast, and released after 15 seconds (s). It was assumed that TI was achieved in birds that did not turn to their right side or get up 10 s after the bird was released. The duration taken by the bird to right or get up was determined by the remote observer with a timer and recorded as the duration of TI. If TI did not occur after 5 repeated TI inductions, the bird was assumed to be sensitive and a score of 0 was given. The test period was limited to a maximum of 10 min, and the duration of TI was taken as 600 s for birds that could not turn to their right side or get up at the end of the procedure (Jones and Faure 1981). TI was assessed by a PhD researcher with vast experience and training in welfare quality assessment protocols.

##### Heterophil/Lymphocyte (H/L) ratio

Blood samples were taken from the wing veins of all the birds tested for TI earlier; 18 broilers (9 males, 9 females) from each litter treatment. Thereafter, a smear was applied on the microscope slide and then stained with the May-Grunwald-Giemsa staining method. After waiting for a certain time, lymphocyte and heterophil cells were counted by magnifying 100X under a light microscope. The percentage of heterophils and lymphocytes obtained for each bird was determined and the H/L ratio was calculated (Gross and Siegel 1983).

##### Proportional asymmetry (PA)

was determined on all the birds that were tested for TI earlier; 9 males and 9 females from each litter treatment. Measurements were determined with a digital caliper (0.01 mm) as described by (Van Nuffel et al. 2007). Measured bilateral traits included third phalanx length of middle toe, fourth phalanx length of outer toe, back toe length, width of tarsometatarsus at the level of the spur, width of tarsometatarsus 1 cm above the spur, joint of tarsometatarsus with tibiotarsus width at the point where it is made, and tarsometatarsus length. PA values were calculated using the formula (|Left-Right|/[(Left + Right)]/2) × 100 (Yang and Siegel 1998).

##### Slaughter and carcass parameters

All the birds that were tested for TI, H/L ratio, and PA were slaughtered at the slaughterhouse of the Gaziosmanpaşa University, Faculty of Agriculture Poultry Unit on 42 d. Feed intake was stopped 12 h before slaughter. The birds were then weighed to determine their slaughter live body weights (SLW). After the slaughtering process, the evisceration process was carried out and the spleen, heart, liver, full gizzard, empty gizzard, digestive system, and abdominal fat were weighed on a scale with a sensitivity of 0.1 g. The internal organ weights were calculated proportionally (g/100 g SLW) according to the following formula (Huang et al. 2009). Proportional internal organ weights (g/100 g SLW) = (Internal organ weight (g) / SLW (g)) × 100.

##### Hot carcass weight (HCW)

was determined with a scale with a precision of 0.1 g. Hot carcass yield (HCY) was calculated using the following formula. HCY, % = (HCW (g) / SLW (g)) × 100.

##### Cold carcass weight (CCW)

was determined after the carcasses whose HCW had been evaluated were kept at + 4 °C for 1d (Bochno et al. 2006). Cold carcass yield (CCY) was calculated using the formula below. CCY, % = (CCW (g) / SLW (g)) × 100.

##### Breast meat pH

was measured from the center at three different regions of the left breast side using a pH meter on the cold carcasses. The readings were obtained by inserting the pH meter at least 3 cm deep into the breast muscle. The average of these three different values was recorded as the pH value (Altan et al. 2001).

##### Carcass portions

involving the breast, drumsticks, wings, back, and neck were obtained from the cold carcasses following the poultry shredding technique of the Turkish Standards Institute (Anonymous 1989). The weights of these portions were determined with a weighing scale of accuracy 0.1 g. Carcass portions were estimated as a percentage of SLW.

### Statistical analyses

Statistical analyses of the data were performed in the SPSS 17.0 statistical package program. Since the number of males and females in the groups was not equal, sex correction was applied to live weights according to the Weighted Mean Difference (TOF) method (Gönül 1974). According to this method, the TOF value was calculated using the following formulas.

$$\begin{array}{l}TO{\rm{F}} = \\\frac{{\left( {{{\bar X}_{11}} - {{\bar X}_{21}}} \right){W_1} + \left( {{{\bar X}_{12}} - {{\bar X}_{22}}} \right){W_2} + \left( {{{\bar X}_{13}} - {{\bar X}_{23}}} \right){W_3}}}{{{W_1} + {W_2} + {W_3}}} \ldots \, \ldots \,..\end{array}$$

$$W=\frac{{n}_{1}. {n}_{2}}{{n}_{1}+{n}_{2}}$$

n_1_; number of males of the respective litter treatment.

n_2_; number of females of the respective litter treatment.

${\overline{\text{X}}}_{11}$; average weight of males in the 1st replicate of the respective litter treatment

${\overline{\text{X}}}_{21}$; average weight of females in the 1st replicate of the respective litter treatment

${\overline{\text{X}}}_{12}$; average weight of males in the 2nd replicate of the respective litter treatment

${\overline{\text{X}}}_{22}$; average weight of females in the 2nd replicate of the respective litter treatment

${\overline{\text{X}}}_{13}$; average weight of males in the 3rd replicate of the respective litter treatment

${\overline{\text{X}}}_{23}$; average weight of males in the 3rd replicate of the respective litter treatment

The TOF value found was divided by two and the resulting value was added to the females, which were removed from the males in the respective litter treatment. Thus, the corrected LW of each individual in a litter treatment was calculated. The same process was applied to other litter treatments and sex correction was made for LW in all groups.

In the study, the normality assumption of the data was examined with Shapiro Wilk, Skewness and Kurtosis tests and it was determined that the normality assumption was met. Additionally, as a result of the Levene test, the variances were homogeneous. For this reason, the following model was used for the variables examined.

$${Y}_{ijk}={\mu +\alpha }_{i}+{\beta }_{j}+{\alpha \beta }_{ij}+{e}_{ijk}$$

In the model ${Y}_{ijk}$: observation value, $\mu$: Population mean ${\alpha}_{i}$: i. Litter type effect, ${\beta}_{j}$: j. Season effect, ${\alpha \beta}_{ij}$:interaction effects and ${e}_{ijk}$: random error.

Mean and common standard error values are given for descriptive statistics values.

Multiple comparisons for variables with significant interaction effects were calculated using one-way analysis of variance. Duncan’s multiple comparison test was used to determine differences between groups. Significance difference was determined at *P* < 0.05. IBM SPSS 21 package program was used in the analysis of all statistical methods (IBM Corp 2012).

## Results

The effect of different litter treatments on LW, FCR, and livability in summer and winter trials is provided in Table 3. On the 21 d, the LW of broilers was similar among the litter groups (*P* > 0.05). However, litter type had a significant effect on 42 d LW (*P* < 0.01). The highest and lowest LW was found in the WS and, BP and WSBP groups, respectively. Additionally, the LW of broilers varied between seasons on 21 and 42 d, with higher LW in winter than in summer (*P* < 0.05).

**Table 3:** Broiler growth performance, carcass yield, and breast meat pH in different litter treatments in summer and winter seasons

	LW, g		FCR, g feed/g LWG		Livability, %
	21 d	42 d	0–21 d	0–42 d	42 d
**Litter treatment (LT)**					
WS	916.86	2560.63^c^	1.38	1.84	97.33
AP	909.18	2516.46^b^	1.39	1.87	97.33
BP	896.70	2454.21^a^	1.39	1.88	98.67
WSAP	910.64	2520.95^bc^	1.40	1.87	97.33
WSBP	908.31	2419.87^a^	1.39	1.88	98.33
**Season (S)**					
Summer	741.44	2117.88	1.34	1.94	98.93
Winter	1078.62	2878.00	1.44	1.80	96.67
**Mean**	908.31	2494.06	1.39	1.87	97.80
**SEM**	4.99	11.97	0.011	0.014	0.47
***P*** **values**					
LT	NS	**	NS	NS	NS
S	**	**	**	**	*
LT × S int	*	**	NS	NS	NS

Abbreviations: WS: wood shavings, AP: acidic pumice stone, BP: basic pumice stone, WSAP: wood shaving + acidic pumice stone (1:1), WSBP: wood shaving + basic pumice stone (1:1), LW: live weight, FCR: feed conversion ratio, d: day, NS: non-significant (*P* > 0.05), SEM: standard error of mean, int: interaction

Means indicated with different letters in the same column are significantly different (*: *P* < 0.05; **: *P* < 0.01)

FCR was similar among litter groups (*P* > 0.05). However, it was observed that FCR from 0 to 21 d was better in the summer and FCR from 0 to 42 d was better in the winter season (*P* < 0.01).

It was identified that litter treatment did not influence livability of broilers (*P* > 0.05). On the other hand, the season had a significant effect on livability of broilers (*P* < 0.05), which was greater in the summer than in the winter season.

Table 4 indicates the results of litter quality variables. A significant difference among litter treatments was observed for litter moisture on the 21 and 42 d (*P* < 0.01). The lowest and highest values were in the BP and WS groups, respectively. In the study, while the effect of the season on litter moisture on the 21 d was found insignificant (*P* > 0.05), the reverse was true on the 42 d (*P* < 0.05). In this study, it was observed that the litter type × season interaction on litter moisture was statistically significant (*P* < 0.05) on the 21 and 42 d.

**Table 4:** Litter quality variables in broilers reared in different litter treatments in the summer and winter seasons

	Moisture, %		pH		Ammonia, mg m^− 2^ s^− 1^	
	21 d	42 d	21 d	42 d	21 d	42 d
**Litter Treatment (LT)**						
WS	24.56^d^	32.01^c^	8.05^a^	8.57	4.07^a^	17.30^a^
AP	6.64^a^	16.06^a^	8.51^c^	8.61	10.23^c^	22.73^b^
BP	6.25^a^	13.60^a^	8.51^c^	8.58	6.37^ab^	25.90^b^
WSAP	19.10^c^	21.02^b^	8.29^b^	8.58	6.63^ab^	16.33^a^
WSBP	11.85^b^	16.06^a^	8.22^ab^	8.57	7.37^b^	23.73^b^
**Season (S)**						
Summer	14.08	17.87	8.45	8.67	5.77	20.75
Winter	13.27	21.40	8.18	8.49	8.09	21.65
**Mean**	13.68	19.75	8.31	8.58	6.93	21.20
**SEM**	1.40	1.41	0.05	0.03	0.60	0.88
***P*** **value**						
LT	**	**	**	NS	**	**
S	NS	**	**	**	**	NS
LT × S int	*	*	NS	NS	*	NS

Abbreviations: WS: wood shavings, AP: acidic pumice stone, BP: basic pumice stone, WSAP: wood shaving + acidic pumice stone (1:1), WSBP: wood shaving + basic pumice stone (1:1), NS: non-significant (*P* > 0.05), SEM: standard error of mean, int: interaction

Means indicated with different letters in the same column are significantly different (*: *P* < 0.05; **: *P* < 0.01)

Although a statistical difference in litter pH was identified among litter treatments on the 21 d (*P* < 0.01), the litter pH was similar on the 42 d (*P* > 0.05). While the highest litter pH value on 21 d was observed in the AP and BP groups, the lowest pH value was in the WS group.

A statistical difference was observed among litter treatments on the 21 and 42 d for litter ammonia (*P* < 0.01). On the 42 d, the highest ammonia was in the BP group, and the lowest was in the WS and WSAP groups. While the litter ammonia varied between seasons on the 21 d (*P* < 0.01), no difference was identified on the 42 d (*P* > 0.05). Again, the influence of litter type × season interaction on litter ammonia was significant on the 21 d (*P* < 0.05) but insignificant on the 42 d (*P* > 0.05).

Table 5 shows the data for litter surface and bottom temperatures for each week. No statistical difference was observed among litter types in terms of litter surface and bottom temperatures (*P* > 0.05). However, there was a significant effect of season on litter temperatures, being higher in summer than in winter season in all weeks (*P* < 0.05; *P* < 0.01), except for week 3 and 6 where the litter bottom temperature in the winter was insignificant (*P* > 0.05).

**Table 5:** Impact of litter materials on weekly litter surface and bottom temperature (°C) in summer and winter

	Litter surface temperature						Litter bottom temperature					
	1. week	2. week	3. week	4. week	5. week	6. week	1. week	2. week	3. week	4. week	5. week	6. week
**Litter treatment (LT)**												
WS	23.64	23.35	28.37	27.12	27.21	26.84	21.93	23.37	29.23	29.30	28.58	28.80
AP	23.32	23.38	28.08	27.50	26.89	26.35	21.88	23.84	29.60	28.49	29.57	28.57
BP	23.10	23.86	28.80	26.59	26.52	26.37	22.67	23.69	29.41	28.13	28.82	28.64
WSAP	23.58	23.95	28.55	27.32	27.30	26.99	22.10	23.50	30.09	29.02	28.74	28.88
WSBP	23.20	23.16	28.36	27.41	26.69	26.13	22.27	23.76	29.52	29.05	28.15	28.25
**Season (S)**												
Summer	26.37	29.29	28.90	29.31	28.55	26.84	25.66	28.30	29.45	30.10	30.76	28.86
Winter	20.37	17.79	27.97	25.07	25.29	26.23	18.68	19.00	29.69	27.49	26.79	28.19
Mean	23.37	23.54	28.43	27.19	26.92	26.53	22.17	23.63	29.57	28.80	28.77	28.63
SEM	0.166	0.169	0.198	0.307	0.176	0.144	0.099	0.111	0.213	0.180	0.199	0.209
***P*** **value**												
LT	NS	NS	NS	NS	NS	NS	NS	NS	NS	NS	NS	NS
S	**	**	*	**	**	*	**	**	NS	**	**	NS
LT × S int	NS	NS	NS	NS	NS	NS	NS	NS	NS	NS	NS	NS

Abbreviations: WS: wood shavings, AP: acidic pumice stone, BP: basic pumice stone, WSAP: wood shaving + acidic pumice stone (1:1), WSBP: wood shaving + basic pumice stone (1:1), NS: non-significant (*P* > 0.05). *: *P* < 0.05; ** *P* < 0.01

Table 6 indicates behavior expression data. There was a significant effect of litter treatment on scratching or foraging behavior during each observation time and the average (*P* < 0.01). The expression of scratching behavior was highest in broilers reared in the AP and lowest in those reared in the BP and WSBP. However, the effect of season and the litter type × season interaction on expression of foraging behavior was found statistically insignificant (*P* > 0.05).

**Table 6:** Behavior expression in broilers (number of birds per minute) reared in different litter treatments in the summer and winter seasons

	Foraging					Dust bathing					Pecking objects				
	20–21 d	27–28 d	34–35 d	41–42 d	Average	20–21 d	27–28 d	34–35 d	41–42 d	Average	20–21 d	27–28 d	34–35 d	41–42 d	Average
**Litter Treatment (LT)**															
WS	2.08^bc^	3.13^b^	1.75^bc^	1.08^b^	2.01^bc^	1.13^bc^	1.79^b^	0.75^bc^	0.42^b^	1.02^b^	5.96	4.67^ab^	6.00	6.46^b^	5.77^abc^
AP	2.46^c^	3.42^b^	2.21^c^	0.96^b^	2.26^c^	1.54^c^	1.58^b^	0.79^bc^	0.46^b^	1.09^b^	6.08	5.58^bc^	6.17	6.79^b^	6.16^bc^
BP	1.17^a^	1.75^a^	0.42^a^	0.17^a^	0.88^a^	0.29^a^	0.37^a^	0.13^a^	0.04^a^	0.21^a^	6.04	5.13^abc^	5.79	5.42^a^	5.59^ab^
WSAP	2.29^a^	2.92^b^	1.33^b^	0.96^b^	1.88^b^	1.42^c^	1.38^b^	0.96^c^	0.50^b^	1.06^b^	5.96	6.04^c^	6.17	6.75^b^	6.23^c^
WSBP	1.54^ab^	1.21^a^	0.42^a^	0.42^a^	0.90^a^	0.67^ab^	0.50^a^	0.37^ab^	0.00^a^	0.39^a^	5.92	4.37^a^	5.79	5.46^a^	5.39^a^
**Season (S)**															
Summer	1.95	2.43	1.13	0.68	1.55	1.13	1.08	0.53	0.30	0.76	3.10	4.42	5.60	5.95	4.77
Winter	1.87	2.53	1.32	0.75	1.62	0.88	1.17	0.67	0.27	0.75	8.88	5.90	6.37	6.40	6.89
**Mean**	1.91	2.48	1.23	0.72	1.58	1.01	1.13	0.60	0.28	0.75	5.99	5.16	5.98	6.18	5.83
**SEM**	0.10	0.15	0.11	0.06	0.06	0.09	0.10	0.07	0.05	0.04	0.30	0.17	0.14	0.12	0.10
***P*** **value**															
LT	**	**	**	**	**	**	**	**	**	**	NS	**	NS	**	**
S	NS	NS	NS	NS	NS	NS	NS	NS	NS	NS	**	**	**	*	**
LT × S int	NS	NS	NS	NS	NS	NS	NS	NS	NS	NS	NS	NS	NS	NS	NS

Abbreviations: WS: wood shavings, AP: acidic pumice stone, BP: basic pumice stone, WSAP: wood shaving + acidic pumice stone (1:1), WSBP: wood shaving + basic pumice stone (1:1), NS: non-significant (*P* > 0.05), SEM: standard error of mean, int: interaction

Means indicated with different letters in the same column are significantly different (*: *P* < 0.05; **: *P* < 0.01)

The effect of litter treatment on expression of dust bathing behavior on all observation days and the general average was identified as significant (*P* < 0.01). On average, the expression of dust-bathing behavior was highest in broilers reared in the AP and lowest in those reared in the BP. On the other hand, there was no effect of season and the litter type × season interaction on expression of scratching behavior on all observation days and the general average (*P* > 0.05).

The effect of litter type on expression of pecking behavior was determined significant on observation d 27–28 and 41–41 (*P* < 0.05), and general average but insignificant on observation days 20–21 and 34–35 (*P* > 0.05). Additionally, it was identified that expression of pecking behavior differed between seasons (*P* < 0.05; *P* < 0.01). In contrast, there was no significant litter type × season interaction effect for the expression of pecking behavior (*P* > 0.05).

The results of FPD, HB, VV, TL, and Bb scores are given in Table 7. It was determined that the litter type and season significantly affected the FPD score in broilers on the 21 and 42 d (*P* < 0.01). On the 21 d, the lowest score was detected in the WS broilers and the highest in the BP and WSBP broilers. On the 42 d, the WS broilers continued to have the lowest score along with the WSAP broilers. BP broilers had the highest FPD score. Furthermore, the FPD score in broilers was higher in the summer than in the winter season. Additionally, a significant litter type × season interaction effect was observed for FPD in broilers on the 21 and 42 d (*P* < 0.01).

**Table 7:** Body region defects in broilers reared in different litter treatments in the summer and winter seasons

	FPD		HB	VV	TL	Bb
	21 d	42 d	42 d	42 d	42 d	42 d
**Litter Treatment (LT)**						
WS	0.60^a^	1.13^a^	0.64^a^	0.000	0.000	0.03^a^
AP	0.90^c^	1.42^b^	1.55^b^	0.004	0.004	0.11^a^
BP	1.56^d^	2.33^d^	2.22^c^	0.003	0.000	1.03^c^
WSAP	0.78^b^	1.23^a^	1.53^b^	0.003	0.000	0.14^a^
WSBP	1.57^d^	2.14^c^	2.24^c^	0.003	0.000	0.56^b^
**Season (S)**						
Summer	1.29	2.01	1.97	0.005	0.003	0.33
Winter	0.87	1.28	1.29	0.000	0.000	0.41
**Mean**	1.08	1.65	1.64	0.003	0.001	0.37
**SEM**	0.02	0.02	0.03	0.000	0.000	0.05
***P*** **value**						
LT	**	**	**	NS	NS	**
S	**	**	**	NS	NS	NS
LT × S int	**	**	**	NS	NS	NS

Abbreviations: WS: wood shavings, AP: acidic pumice stone, BP: basic pumice stone, WSAP: wood shaving + acidic pumice stone (1:1), WSBP: wood shaving + basic pumice stone (1:1), FPD: footpad dermatitis, HB: hock burn, VV: varus-valgus, TL: twisted leg, Bb: breast blister, NS: non-significant (*P* > 0.05), SEM: standard error of mean, int: interaction

Means indicated with different letters in the same column are significantly different (*: *P* < 0.05; **: *P* < 0.01)

Also, variation was observed regarding the HB score in broilers among litter types and between seasons on the 42 d (*P* < 0.01). The lowest score was detected in the WS broilers and the highest in the BP and WSBP broilers. Likewise, the HB score in broilers was higher in the summer than in the winter season. Moreover, a significant litter type × season interaction effect was identified for HB score in broilers on the 42 d (*P* < 0.01).

There were no differences in VV and TL occurrence in broilers among litter treatments, between seasons, and their interactions (*P* > 0.05).

It was found that only litter treatment significantly influenced Bb score (*P* < 0.05), highest and lowest for BP and, WS, AP, and WSAP broilers, respectively.

The results of body region temperatures are shown in Table 8. The effect of litter type on rectal, footpad, and breast surface temperature in broilers on d 21 and 42 was found insignificant (*P* > 0.05), except for breast surface temperature on 42 d (*P* < 0.05). The breast surface temperature was lowest in WS and AP broilers, and highest in BP and WSBP broilers. Additionally, except for the 21 d rectal temperature, the seasonal effect was observed significant for all the body region temperatures (*P* < 0.01), higher in the broilers in the summer trial than those in the winter trial.

**Table 8:** Body region temperatures (°C) in broilers raised in different litter types in summer and winter

	Rectal temperature		Footpad temperature		Breast region surface temperature	
	21 d	42 d	21 d	42 d	21 d	42 d
**Litter Treatment (LT)**						
WS	41.18	41.38	34.08	29.52	35.87	34.58^a^
AP	40.49	41.33	32.58	29.04	35.67	34.52^a^
BP	41.21	41.31	33.04	29.35	35.70	36.06^b^
WSAP	41.17	41.36	33.09	30.21	35.74	35.92^b^
WSBP	41.18	41.35	32.18	29.93	36.32	35.14^ab^
**Season (S)**						
Summer	40.96	41.52	33.95	31.70	37.96	36.79
Winter	41.12	41.17	32.04	27.51	34.26	33.70
**Mean**	41.04	41.35	32.99	29.61	35.86	35.25
**SEM**	0.17	0.03	0.24	0.27	0.18	0.20
***P*** **value**						
LT	NS	NS	NS	NS	NS	*
S	NS	**	**	**	**	**
LT × S int	NS	NS	NS	NS	NS	**

Abbreviations: WS: wood shavings, AP: acidic pumice stone, BP: basic pumice stone, WSAP: wood shaving + acidic pumice stone (1:1), WSBP: wood shaving + basic pumice stone (1:1), NS: non-significant (*P* > 0.05), SEM: standard error of mean, int: interaction

Means indicated with different letters in the same column are significantly different (*: *P* < 0.05; **: *P* < 0.01)

Table 9 indicates the results of FCS, TI, and H/L ratio. The effect of the type of litter on the back and breast cleanliness of broilers on the 21 and 42 d was found significant (*P* < 0.01). Generally, WS and, BP and WSBP broilers had cleaner and dirtier back and breast feathers, respectively. Also, there was a significant seasonal effect on back and breast feather cleanliness score (*P* < 0.01). It was determined that broilers in the summer trial had dirtier back and breast feathers than those in the winter season on the 21 and 42 d. Furthermore, the season × litter type interaction significantly affected the back and breast cleanliness score of broilers on the 21 and 42 d (*P* < 0.01).

**Table 9:** Feather cleanliness score, tonic immobility duration, and heterophil/lymphocyte ratio of broiler chickens raised on different litter types in summer and winter seasons

	Back FCS		Breast FCS		TI duration, seconds	H/L ratio
	21 d	42 d	21 d	42 d	42 d	42 d
**Litter Treatment (LT)**						
WS	1.15^a^	1.47^a^	1.86^a^	2.59^a^	213.27	0.58
AP	1.15^a^	1.43^a^	1.84^a^	2.70^b^	206.14	0.59
BP	1.43^c^	2.38^c^	1.86^a^	2.92^c^	279.81	0.61
WSAP	1.21^a^	1.63^b^	1.86^a^	2.68^b^	178.87	0.59
WSBP	1.29^b^	2.39^c^	2.05^b^	2.93^c^	221.90	0.60
**Season (S)**						
Summer	1.44	1.92	2.46	2.78	216.55	0.57
Winter	1.04	1.81	1.32	2.75	223.45	0.62
**Mean**	1.24	1.87	1.90	2.76	220.00	0.59
**SEM**	0.01	0.02	0.02	0.01	13.19	0.01
***P*** **value**						
LT	**	**	**	**	NS	NS
S	**	**	**	NS	NS	*
LT × S int	**	**	**	**	NS	NS

Abbreviations: WS: wood shavings, AP: acidic pumice stone, BP: basic pumice stone, WSAP: wood shaving + acidic pumice stone (1:1), WSBP: wood shaving + basic pumice stone (1:1), NS: non-significant (*P* > 0.05), SEM: standard error of mean, int: interaction, FCS: feather cleanliness scores, TI: tonic immobility, H/L ratio: Heterophil-to-Lymphocyte ratio

Means indicated with different letters in the same column are significantly different (*: *P* < 0.05; **: *P* < 0.01)

The litter type, season, and their interaction did not affect the duration of TI (*P* > 0.05).

Meanwhile, the H/L ratio was similar among litter types (*P* > 0.05) but dissimilar between seasons (*P* < 0.05), higher in broilers in the winter than those in the summer trial. However, the interaction between litter type × season did not affect the H/L ratio (*P* > 0.05).

Table 10 shows data for the PA of broilers. It was observed that the PA of bilateral traits was not different among the litter groups (*P* > 0.05). On the other hand, PA of the bilateral traits varied between seasons (*P* < 0.05; *P* < 0.01), except for tarsometatarsus width at spur level and tarsometatarsus length (*P* > 0.05). Likewise, there was no litter type × season interaction effect on PA (*P* > 0.05).

**Table 10:** Proportional asymmetry of broiler on 42 d raised in different litter types in summer and winter seasons

	D	E	F	G	H	I	J
**Litter Treatment (LT)**							
WS	3.96	4.29	4.09	3.46	2.74	2.80	2.26
AP	4.09	6.02	3.92	3.77	3.08	2.77	2.21
BP	4.62	5.69	5.14	4.03	2.87	3.80	2.39
WSAP	4.23	6.29	4.39	3.48	2.43	3.14	2.28
WSBP	4.17	5.68	4.26	4.56	3.48	2.99	2.28
**Season (S)**							
Summer	5.03	6.68	5.16	3.89	3.46	3.75	2.58
Winter	3.39	4.50	3.56	3.83	2.38	2.45	1.98
**Mean**	4.21	5.59	4.36	3.86	2.92	3.10	2.28
**SEM**	0.23	0.31	0.26	0.22	0.18	0.32	0.16
***P*** **value**							
LT	NS	NS	NS	NS	NS	NS	NS
S	**	**	**	NS	**	*	NS
LT × S int	NS	NS	NS	NS	NS	NS	NS

Abbreviations: WS: wood shavings, AP: acidic pumice stone, BP: basic pumice stone, WSAP: wood shaving + acidic pumice stone (1:1), WSBP: wood shaving + basic pumice stone (1:1), NS: non-significant (*P* > 0.05), SEM: standard error of mean, int: interaction, D: middle toe 3rd phalanx length, E: length of outer toe 4th phalanx, F: back toe length, G: tarsometatarsus width at spur level, H: tarsometatarsus width from 1 cm above the spur, I: width of the tarsometatarsus where it joins the tibiotarsus joint, J: tarsometatarsus length,

Means indicated with different letters in the same column are significantly different (*: *P* < 0.05; **: *P* < 0.01)

The results of HCY, CCY, breast meat pH, carcass portions and proportional internal organ weights are shown in Table 11. The effect of different litter treatments on HCY and CCY of broilers was found significant (*P* < 0.01; *P* < 0.05). The lowest and highest yields were observed in the WSBP and WS groups, respectively. It was determined that the effect of season on HCY was significant (*P* < 0.01) but nonsignificant for CCY (*P* > 0.05). Statistically, HCY was higher in the summer than in the winter season. Furthermore, it was observed that the effect of litter treatment and season on breast meat pH was insignificant (*P* > 0.05).

**Table 11:** Carcass traits, carcass portions (%), and proportional internal organ weights (g/100 g LW) in broilers reared in different litter treatments in the summer and winter seasons

	HCY, %	CCY, %	Breast meat pH	Breast	Drumsticks	Wings	Back	Neck	Spleen	Heart	Liver	Empty gizzard	Digestive tract	Abdominal fat
**Litter Treatment (LT)**														
WS	74.08^b^	72.59^b^	6.09	35.21	28.35	9.71^ab^	20.69	6.05^a^	0.15^a^	0.59^a^	1.99^ab^	1.51	4.67	1.52^a^
AP	72.42^a^	71.18^ab^	6.12	35.52	27.85	9.45^a^	20.62	6.52^b^	0.16^a^	0.59^a^	1.90^a^	1.46	4.64	1.71^b^
BP	72.16^a^	71.07^ab^	6.08	36.24	27.52	9.46^a^	20.36	6.41^b^	0.17^b^	0.65^b^	2.12^c^	1.55	4.85	1.47^a^
WSAP	72.64^ab^	71.38^ab^	6.06	35.73	27.86	9.50^a^	20.34	6.53^b^	0.15^a^	0.60^a^	2.00^ab^	1.50	4.65	1.55^ab^
WSBP	71.29^a^	70.04^a^	6.15	35.52	27.68	9.95^b^	20.06	6.76^b^	0.16^ab^	0.65^b^	2.08^bc^	1.52	4.75	1.40^a^
**Season (S)**														
Summer	73.32	71.62	6.11	34.71	28.77	10.13	20.44	5.90	0.22	0.73	2.31	2.01	5.97	1.51
Winter	71.72	70.89	6.09	36.58	26.93	9.10	20.38	7.01	0.10	0.50	1.73	1.01	3.46	1.55
**Mean**	72.52	71.25	6.10	35.64	27.85	9.61	20.41	6.45	0.16	0.62	2.02	1.51	4.71	1.53
**SEM**	0.25	0.24	0.01	0.15	0.12	0.06	0.10	0.07	0.005	0.011	0.027	0.040	0.105	0.029
***P*** **value**														
LT	**	*	NS	NS	NS	**	NS	**	**	**	**	NS	NS	**
S	**	NS	NS	**	**	**	NS	**	**	**	**	**	**	NS
LT × S int	NS	NS	NS	NS	NS	**	NS	**	NS	NS	**	**	NS	**

Abbreviations: LW: live weight, NS: non-significant (*P* > 0.05), SEM: standard error of mean, int: interaction, WS: wood shavings, AP: acidic pumice stone, BP: basic pumice stone, WSAP: wood shaving + acidic pumice stone (1:1), WSBP: wood shaving + basic pumice stone (1:1), HCY: hot carcass yield, CCY: cold carcass yield,

Means indicated with different letters in the same column are significantly different (*: *P* < 0.05; **: *P* < 0.01)

It was identified that litter treatment affected wing and neck ratios (*P* < 0.05). However, the breast, drumstick, and back ratios were similar among litter treatments (*P* > 0.05). Except for the back ratio (*P* > 0.05), the effect of season on the remaining carcass portion ratios was found significant (*P* < 0.01). While the effect of litter type on relative spleen, heart, liver, and abdominal fat weight was determined significant (*P* < 0.01), it was the opposite for empty gizzard and digestive tract weights (*P* > 0.05). There was a significant seasonal effect on internal organ weights (*P* < 0.01) excluding abdominal fat. The weights of the affected internal organs were greater in summer than in the winter season.

## Discussion

The present findings indicated that litter types varied regarding the LW of broilers, which comply with the results of some studies (Sarıca and Çam 2000; Eleroğlu and Yalçın 2005; Petek et al. 2014; Garcês et al. 2017). However, they contradict Villagrá et al. (2011), Durmuş et al. (2023), and Şen et al. (2023) who reported similar LW of broilers on the 42 d in different litter materials. It is argued that the inconsistencies among studies might be due to differences in genotypes, production conditions, litter materials, study region, etc.

The quality of all the litter materials was quite high at the beginning of the experiments. Wood shavings can sustain such a high quality for a long time, leading to greater LW. However, BP was easily spoiled in a short time, resulting in lower LW and it has been reported that a reduction in the quality of litter reduces the live weight gains (Weeks and Butterworth 2004). Also, BP has sharp and hard edges or corners that may irritate the animals. Furthermore, BP has a less porous structure thus, a less water absorption capacity (Yaşar and Erdoğan 2005). Because of all these characteristics, the quality of BP was assessed as poor and an earlier reduction in quality was also observed in WSBP, which was linked to lower LWG in broilers reared in these litter materials. On the other hand, AP has rounded and soft edges or corners, with quite a high water absorption capacity (53.05%) (Yaşar and Erdoğan 2005). Generally, broilers reared in AP and WSAP litter materials had LWG close to WS broilers.

The lower LW of broilers in the summer trial could be linked to the higher ambient temperatures and increased activity of birds in the summer season. However, regardless of the season, broilers reared in WS had the highest LW, indicating that WS can be considered as the best litter material for broilers.

In the current study, the effect of litter type on FCR between 0 and 42 d complies with the findings of several studies, which found similar FCR of broilers in different litter materials (Sarıca and Çam 2000; Villagrá et al. 2011; Petek et al. 2014; Şen et al. 2023). In contrast, some authors reported dissimilar FCR of broilers reared in different litter types (Monira et al. 2003; Eleroğlu and Yalçın 2005; Garcês et al. 2017; Durmuş et al. 2023). The differences in study findings are mostly due to factors including differences in genotypes, litter materials, study region, and production conditions.

The effect of litter material × season interaction on FCR was found insignificant as observed by Petek et al. (2014). Therefore, it can be speculated that the use of different litter materials in different seasons does not influence the pattern of changes in the FCR of broilers.

The results of the present study are in line with several studies that showed no variations in mortality rate in broilers reared in different litter materials (Eleroğlu and Yalçın 2005; Durmuş et al. 2023). However, the opposite pattern was observed in other reports (Sarıca and Çam 2000; Garcês et al. 2017).

In the present study, litter moisture content, pH, and ammonia varied among the litter types. This is in agreement with some studies that found differences among litter materials in terms of litter quality parameters. For instance, Garcês et al. (2013) identified a higher moisture percentage in coconut husk than in wood shavings. Furthermore, the moisture percentage of rice hulls, newspaper, and corn cob litters was comparable to that of wood shavings. Petek et al. (2014) reported a higher moisture percentage in wood shaving than in rice hull litter material.

Regarding ammonia concentration, Garcês et al. (2017) reported a greater ammonia concentration in guinea grass than in wood shaving whose value was lower than that of sand and newspaper. Also, Garcês et al. (2013) found a higher ammonia concentration in sand, guinea grass, and newspaper than in wood shavings. Moreover, for litter pH, Petek et al. (2014) observed a greater pH value for rice hull than wood shaving.

In contrast to the findings of the present study, Şen et al. (2023) observed no differences between wood shaving and barley straw either used alone or in a mixed ratio in terms of moisture content and ammonia level. Also, Garcês et al. (2013) did not find differences among river bed sand, coconut husk, rice hull, guinea grass, newspaper combined with wood shavings, and corn cob as litter materials regarding the litter pH.

Furthermore, Bist et al. (2023) reported that ammonia gas dominates the environment when the pH is 7 and above. Therefore, ammonia emission can be reduced by ensuring that the litter pH shifts to acid (Ashtari et al. 2016; Chai et al. 2017). Also, Tan et al. (2019) confirmed that some organic materials possess ammonia-inhibiting properties, ensuring better litter quality materials. Based on these facts, it can be argued that AP and WSAP are a better substrate than BP.

Regarding the season effect, the moisture content of litter in the winter trial was higher than that in the summer trial on the 42 d. This was likely due to the higher ambient temperatures in summer, which resulted in increased evaporation of moisture from litter and consequently less moisture content. In addition, the pH of litter in the summer was higher than that of the winter trial. This could be associated with the fact that the litter pH might have shifted towards alkalinity due to excessive litter moisture in winter as suggested by Bist et al. (2023).

The effect of litter type × season interaction on litter quality variables based on sampling day might indicate the pattern of changes in these variables due to the differences in water holding and retention capacity of different litter types in different seasons.

In the current study, the lack of litter type effect on litter temperature is not in line with Garcês et al. (2017), who reported a higher litter surface temperature of coconut husks than WS, sand, rice hulls, guinea grass, newspaper, and corn cob, whose values were similar. Garcês et al. (2017) emphasized that the results were linked to the fact that coconut husks have lower thermal conductivity and are a better insulator than the other substrates. Based on these arguments, the variations between their results and the present study might be related to the differences in the litter materials. Additionally, other factors including genotype, housing conditions, management of birds, and study region could also result in differences among studies. It can also be argued to undertake further studies to determine the impact of litter type on litter temperature.

The influence of season on litter temperature is probably due to the ambient temperature. For instance, due to the higher ambient temperature in summer, the rate of moisture evaporation from the litter will increase causing it to be dry; however, the winter season comes with a humid environment which in turn increases the moisture content of litter. This decreases the litter temperature.

The small size particles and relatively loose litter materials are important factors associated with the motivation of birds to express litter-directed behaviors including dust bathing, foraging, and pecking (Evans et al. 2023; Holt et al. 2023; Vas et al. 2023). In this study, dust bathing, ground scratching, and pecking of objects (averages) were expressed mostly in AP litter, followed by WS and WSAP litter which could be associated with AP particles being round, soft, and easily broken natural stone. These litter materials could be turned easily by the birds. On the other hand, hard and sharp-edged particles are probable reasons for decreased litter-directed behaviors in BP and WSBP litter. The impact of litter type on the expression of litter-directed behaviors has also been reported by Holt et al. (2023).

In the poultry industry, contact dermatitis including FPD, HB, and Bb is considered as an indicator of broiler health and welfare. Their occurrence has been mainly linked to severe pain, and reduced feed intake and weight gain due to induced pain, lameness, etc., (Bessei 2006; Tainika et al. 2023; Dawkins 2024). Studies have confirmed a high incidence of FPD in broilers reared in wet and hard litter (Dawkins et al. 2004; Weeks and Butterworth 2004).

Indeed, in the present study, a link was observed between poor litter quality and the high occurrence of FPD, HB, and Bb. BP and WSBP had higher litter moisture percentages and BP was characterized by hard and sharp edges, which could harm the feet of broilers. These negative properties could be the most likely reasons behind the most severe FPD, HB, and Bb cases identified in the BP and WSBP broilers. Additionally, there are some reports indicating differences in FPD scores (Petek et al. 2014; Garcês et al. 2017; Durmuş et al. 2023), HB scores (Weeks and Butterworth 2004; Villagrá et al. 2011), and Bb scores (Weeks and Butterworth 2004) in broilers reared in different litter materials. On the other hand, other authors reported that the occurrence of HB (Garcês et al. 2017) and Bb (Villagrá et al. 2011) did not differ in broilers reared in different litter materials.

In addition, it was observed that the FPD and HB scores detected in broilers in the summer trial were higher than in the winter season. Again, this finding can be associated with poor litter quality in the winter season.

Furthermore, the effect of litter type × season interaction on FPD and HB scores might indicate the pattern of changes in these variables as litter quality deteriorates in different seasons.

In the present study, VV deformation and TL syndrome were not observed. These results are in agreement with Barbosa et al. (2022), who reported that the use of different litter types did not affect VV deformity in broilers. Also, Paz et al. (2013) showed no differences in VV deformity in broilers reared on rice husks and WS. Additionally, Vargas-Galicia et al. (2017) reported that tezontle (volcanic rock) and WS litter did not differ in terms of VV deformity in broilers. In contrast, Khan et al. (2023) found a higher incidence of VV deformity in broilers reared in plastic-grid flooring than in zeolite-added litter and WS.

In this study, the differences in FCS among the litter types and seasons and their interaction effect are associated with the pattern of changes in litter quality in different litter types and seasons. Indeed, BP and WSBP litter materials were wetter and most soiled during the trials. This corresponds to the lower back and breast FCS of broilers reared in BP and WSBP observed in this study.

In the current study, body region temperatures were similar among the litter types except breast region temperature on the 42 d. Chickens are homeothermic animals, having the ability to regulate their body temperature by using their physiological and behavioral mechanisms to resist the negative effects of the environment. Based on the above facts, the current results can somehow be expected. Contrary to the findings of the present study, Garcês et al. (2017) reported higher footpad surface temperature for broilers reared in coconut husk than those reared in WS, sand, rice hull, newspaper, and corn cob. It is suggested that the influence of litter type on body region temperatures needs further studies. This is because Garcês et al. (2017) associated lower footpad surface temperatures with a negative impact on broiler performance in guinea grass, which was not found in other litter groups.

In the present study, the seasonal effect on body region temperatures might reflect the pattern of changes in the bird’s physiological response to changes in environmental temperatures.

The H/L ratio is a physiological indicator of the response of animals to stress. The H/L ratio is a reliable indicator and when taken as a reference value, 0.2 is considered to be low stressful, 0.5 is medium and 0.8 is high stressful (Gross and Siegel 1983). In this study, H/L ratios were not different among litter types and were between 0.58 and 0.61 in different litter types. Thus, it can be argued that the stress level of the chickens was at a moderate level. Furthermore, the impact of litter types on H/L ratios has not been fully investigated. However, Huang et al. (2009) reported that litter type influenced red blood cell count in birds but did not affect the percentage of white blood cells and lymphocytes.

Also, the duration of TI is a well-known physiological indicator to assess fearfulness in birds (Jones and Faure 1981). In the current study, the duration of TI did not vary among the litter treatments, which is in line with the findings of Villagrá et al. (2011), when they used WS and paper residue litters. Also, Avcılar et al. (2018) did not find a significant difference between WS and rice hull regarding the duration of TI.

In the present study, the PA of bilateral traits was similar among litter materials. Therefore, it can be concluded that pumice alone or in combination with WS does not have any negative effect on PA in broiler chickens. Nevertheless, the results of the present study are consistent with Villagrá et al. (2011), who reported that fluctuating asymmetry (cm) did not differ in broilers reared on WS and paper residue litter. On the other hand, the larger PA of some bilateral traits in broilers in the summer trial than in the winter trial could indicate that heat stress probably due to the higher ambient temperature might result in the developmental instability of these traits.

However, although there is limited evidence regarding the influence of different litter types on PA, other environmental factors including production system (Knierim et al. 2007; Tuyttens et al. 2008), stocking density (Buijs et al. 2012), and type of plant species in outdoor areas of the free-range system (Bashir et al. 2023) have been suggested to modulate the PA of some bilateral traits.

In the current study, it was observed that litter type influenced the carcass traits, with the exceptions being back weight and breast meat pH. This is inconsistent with the previous studies that found no difference in carcass parameters including hot carcass weight, carcass yield, breast meat, wing, leg portion, and liver as a percentage of LW in broilers reared in different litter types (Toghyani et al. 2010; Garcês et al. 2017; Şen et al. 2023; Durmuş et al. 2023). Again, the sources of variation in the studies might originate from factors such as the differences in litter materials, broiler genotypes, housing conditions, bird management, and study region. Furthermore, it can be argued that the qualities of BP and WSBP might adversely impact the biological activities in broilers, which modify the physiological responses. This might result in detrimental effects on performance and carcass traits.

In the present study, breast meat pH was similar among the litter types, which is in line with the findings of Durmuş et al. (2023). Additionally, the breast meat pH of broilers reared in different litter materials varied between 6.06 and 6.15, which was slightly above the broiler meat pH range under normal conditions (Petracci et al. 2015). Generally, low pH indicates better animal welfare before slaughter and provides advantages during processing (Castellini et al. 2002). Notably, there is limited literature about the effect of different litter materials on breast meat pH, which warrants further studies. However, factors including preslaughter handling, slaughter process, and nutrition can influence broiler meat pH (Petracci et al. 2015; Mir et al. 2017).

Except for HCY, CCY, breast meat pH, back weight, and abdominal fat, the season had a significant effect on other carcass variables. The affected variables were higher in broilers in summer than those in winter. This is probably linked to lower carcass weights of broilers in summer than those in winter season. In both seasons, broilers reared in WS had the best carcass yields, emphasizing its suitability in broiler production.

The effect of litter type × season interaction on some carcass variables might indicate the pattern of changes in these variables as broilers respond to different litter types and seasons that come with maturity.

## Conclusions

This study was able to determine that WS is still the best litter material, owing to superiority, especially in terms of LW, litter moisture content and ammonia concentration, body defects (FPD, HB, Bb), breast and back FCS, and carcass efficiency. AP and WSAP were superior to WS in terms of motivating birds to express litter-directed behaviors as decreasing the duration of TI in birds. In addition, AP results are close to WS in terms of performance traits properties. However, BP is associated with adverse impacts on LW, litter quality, CCY, body defects, FCS, and behavior expression. Therefore, this study suggests that AP alone or in combination with WS can be easily used as an alternative litter resource. This is because it does not adversely affect the performance and welfare of broilers.

### Electronic supplementary material

Below is the link to the electronic supplementary material.

Supplementary Material 1

## Data Availability

Not applicable.
