# Supplementary material for: The potential of pumice as a litter material and its influence on growth performance, carcass parameters, litter quality traits, behavior, and welfare in broiler chickens
Source: Trop Anim Health Prod. 2024 Apr 18;56(4):130. doi: 10.1007/s11250-024-03979-z (PMC11026241; doi:10.1007/s11250-024-03979-z)
Supplement: Supplementary file 1 — Supplementary Material 1 [file 11250_2024_3979_MOESM1_ESM.docx]

Supplementary Table 1 Number of males and females per litter treatment replicate on the initial and final day of the study trials

|  |  |  | Day 1 | | | Day 42 | | |
| --- | --- | --- | --- | --- | --- | --- | --- | --- |
|  | Litter treatment | Sex | R1 | R2 | R3 | R1 | R2 | R3 |
| Summer | WS | Male | 17 | 20 | 19 | 17 | 18 | 18 |
|  |  | Female | 33 | 30 | 31 | 33 | 29 | 31 |
|  | AP | Male | 25 | 19 | 21 | 25 | 19 | 20 |
|  |  | Female | 25 | 31 | 29 | 25 | 31 | 29 |
|  | BP | Male | 26 | 20 | 15 | 26 | 20 | 15 |
|  |  | Female | 24 | 30 | 35 | 24 | 30 | 35 |
|  | WSAP | Male | 27 | 18 | 26 | 27 | 15 | 26 |
|  |  | Female | 23 | 32 | 24 | 23 | 32 | 24 |
|  | WSBP | Male | 16 | 27 | 16 | 16 | 27 | 16 |
|  |  | Female | 34 | 23 | 34 | 34 | 23 | 34 |
| Winter | WS | Male | 27 | 23 | 29 | 27 | 23 | 29 |
|  |  | Female | 23 | 27 | 21 | 21 | 27 | 19 |
|  | AP | Male | 28 | 26 | 22 | 28 | 22 | 22 |
|  |  | Female | 22 | 24 | 28 | 22 | 23 | 26 |
|  | BP | Male | 20 | 20 | 23 | 19 | 18 | 22 |
|  |  | Female | 30 | 30 | 27 | 30 | 30 | 27 |
|  | WSAP | Male | 21 | 24 | 24 | 21 | 22 | 24 |
|  |  | Female | 29 | 26 | 26 | 27 | 25 | 26 |
|  | WSBP | Male | 26 | 29 | 18 | 26 | 27 | 18 |
|  |  | Female | 24 | 21 | 32 | 22 | 21 | 31 |

Supplementary Table 2 Weekly average temperature values inside the barn (at head level) and outside the barn in different litter types in summer and winter, °C

| Season | Litter treatment | 1. week | 2. week | 3. week | 4. week | 5. week | 6. week |
| --- | --- | --- | --- | --- | --- | --- | --- |
| Summer | WS | 29.32 | 26.99 | 29.21 | 27.38 | 26.98 | 24.79 |
|  | AP | 28.27 | 26.96 | 29.34 | 27.56 | 26.76 | 25.44 |
|  | BP | 28.34 | 26.89 | 29.12 | 27.52 | 26.82 | 25.34 |
|  | WSAP | 28.48 | 27.13 | 29.39 | 27.67 | 26.98 | 25.64 |
|  | WSBP | 28.80 | 27.13 | 29.37 | 27.70 | 27.15 | 25.48 |
|  | P | NS | NS | NS | NS | NS | NS |
| Winter | WS | 26.13 | 21.87 | 20.60 | 23.50 | 24.51 | 21.79 |
|  | AP | 26.26 | 21.90 | 20.17 | 23.49 | 25.49 | 22.28 |
|  | BP | 28.33 | 23.45 | 21.77 | 24.73 | 26.22 | 22.15 |
|  | WSAP | 26.56 | 22.08 | 20.49 | 23.36 | 25.29 | 21.45 |
|  | WSBP | 26.32 | 22.15 | 20.17 | 22.86 | 24.76 | 21.85 |
|  | P | NS | NS | NS | NS | NS | NS |
| Outside the barn in summer | | 24.98 | 25.73 | 29.28 | 26.72 | 25.41 | 23.69 |
| Outside the barn in winter | | 3.99 | 0.18 | 1.77 | 8.67 | 8.85 | 8.21 |

Abbreviations: WS: wood shavings, AP: acidic pumice stone, BP: basic pumice stone, WSAP: wood shaving + acidic pumice stone (1:1), WSBP: wood shaving + basic pumice stone (1:1), NS: non-significant (P>0.05)

Supplementary Table 3 Weekly average humidity values inside the barn (at head level) and outside the barn in different litter types in summer and winter, %

| Season | Litter treatment | 1. week | 2. week | 3. week | 4. week | 5. week | 6. week |
| --- | --- | --- | --- | --- | --- | --- | --- |
| summer | WS | 47.13^a^ | 52.91^ab^ | 52.37^ab^ | 56.40^b^ | 57.58^ab^ | 56.28^ab^ |
|  | AP | 46.32^a^ | 51.70^a^ | 50.28^a^ | 53.85^a^ | 55.86^a^ | 54.22^a^ |
|  | BP | 48.08^a^ | 53.31^ab^ | 52.55^ab^ | 56.75^b^ | 59.04^bc^ | 57.15^b^ |
|  | WSAP | 49.98^ab^ | 55.17^b^ | 54.41^b^ | 58.55^b^ | 60.21^c^ | 58.53^b^ |
|  | WSBP | 56.78^b^ | 62.21^c^ | 61.84^c^ | 66.28^c^ | 66.94^d^ | 66.90^c^ |
|  | P | * | ** | ** | ** | ** | ** |
| Winter | WS | 29.74^b^ | 39.03^d^ | 47.23^c^ | 43.35^d^ | 53.15^d^ | 60.00^c^ |
|  | AP | 34.84^c^ | 43.58^e^ | 53.21^d^ | 47.99^e^ | 55.90^d^ | 64.15^c^ |
|  | BP | 23.35^a^ | 27.27^a^ | 32.50^a^ | 29.35^a^ | 33.20^a^ | 37.28^a^ |
|  | WSAP | 25.17^a^ | 30.35^b^ | 35.98^a^ | 33.60^b^ | 40.91^b^ | 50.75^b^ |
|  | WSBP | 26.78^ab^ | 33.01^c^ | 41.76^b^ | 37.95^c^ | 46.09^c^ | 51.97^b^ |
|  | P | ** | ** | ** | ** | ** | ** |
| Outside the barn in summer | | 57.47 | 58.15 | 51.84 | 57.24 | 60.26 | 58.36 |
| Outside the barn in winter | | 79.86 | 92.96 | 79.17 | 58.78 | 76.16 | 66.93 |

Abbreviations: WS: wood shavings, AP: acidic pumice stone, BP: basic pumice stone, WSAP: wood shaving + acidic pumice stone (1:1), WSBP: wood shaving + basic pumice stone (1:1), Means indicated with different letters in the same column are significantly different (*: P<0.05; **: P<0.01).
